# Effect of Gelatin–Peptide Complex from Sturgeon Skin on Behavioral, Antioxidant, and Neuroprotective Functions in D-Galactose-Induced Aging Mice: Thermal Degradation vs. Enzymatic Hydrolysis

**DOI:** 10.3390/foods15101624

**Published:** 2026-05-07

**Authors:** Siyuan Ma, Yibing He, Ying Han, Wei Zhao, Hanxue Sun, Zhenyu Wang, Yiying Nian, Peng Liu, Ming Du, Liming Sun

**Affiliations:** 1 SKL of Marine Food Processing & Safety Control, National Engineering Research Center of Seafood, School of Food Science and Technology, Dalian Polytechnic University, Dalian 116034, China; 231720860001090@xy.dlpu.edu.cn (S.M.); hyb1730393764@163.com (Y.H.); hy17584566660@163.com (Y.H.); 13352233260@163.com (W.Z.); 18145685500@163.com (H.S.); wangzhenyu@dlpu.edu.cn (Z.W.); duming@dlpu.edu.cn (M.D.); 2 Institute of Agriculture and Food Standardization, China National Institute of Standardization, Beijing 100191, China; nianyy@cnis.ac.cn (Y.N.); liupeng@cnis.ac.cn (P.L.)

**Keywords:** sturgeon skin, gelatin-peptide complex, thermal degradation, enzymatic hydrolysis, anti-aging effects

## Abstract

Collagen-derived products are widely applied in functional foods; however, limited information is available regarding how different preparation methods, particularly thermal degradation and enzymatic hydrolysis, affect their anti-aging efficacy and biological functions. In this study, sturgeon skin was used as raw material to prepare gelatin–peptide complexes via thermal degradation (GPC-TD) and enzymatic hydrolysis (GPC-EH), and their comparative anti-aging and biological effects were evaluated in D-galactose-induced aging mice. Female ICR mice were divided into eight groups: a blank control group (normal saline), an aging model group (D-galactose, 500 mg/kg), three GPC-TD and three GPC-EH groups (D-galactose supplemented with 100, 200, 400 mg/kg GPC-TD or GPC-EH). After eight weeks of administration, various physiological parameters were evaluated. Throughout the experiment, no statistically significant difference in body weight (BW) was observed among the groups; however, the blank and model groups consistently maintained the highest BW. The medium- and high-dose GPC-TD groups showed relatively faster weight gain, whereas the 100 mg/kg GPC-TD group and all three GPC-EH groups exhibited the slowest BW gain. Notably, the gastric indices of these latter groups were significantly lower than those of other groups (*p* < 0.05), which might be a key factor affecting BW gain. Behavioral tests revealed that the model group exhibited significantly reduced swimming speed and weakened nesting ability (*p* < 0.05), both of which were alleviated to varying degrees by treatment with GPC-TD and GPC-EH. Furthermore, both complexes markedly decreased malondialdehyde content in liver tissue (*p* < 0.05). Compared with the model group, high-dose GPC-TD and GPC-EH effectively increased acetylcholine content and inhibited acetylcholinesterase activity (*p* < 0.05). Masson staining revealed abnormal collagen fibers accumulation in certain tissues of model mice, a condition that was clearly ameliorated by GPC-TD and, to a greater extent, by GPC-EH. In addition, medium and high doses of both complexes significantly protected against D-galactose-induced loss of Nissl bodies in brain neurons; in the high-dose GPC-EH group, the density and number of Nissl bodies approached those observed in the blank group. These findings suggest that both GPC-TD and GPC-EH possess potential anti-aging effects, with GPC-EH exhibiting superior efficacy. This study provides theoretical support for consumers, the catering industry, and manufacturers in selecting appropriate processing techniques for the preparation of sturgeon skin GPC.

## 1. Introduction

Population aging is accelerating at an unprecedented pace [1], drawing global attention to aging-related healthcare and effective intervention strategies [2]. Aging is a multifactorial biological process involving the gradual buildup of molecular and cellular damage, ultimately resulting in the deterioration of tissue and organ functions [3,4]. Collagen is the most abundant structural protein in mammals and plays a key role in maintaining the integrity and mechanical properties of the extracellular matrix (ECM) [5,6]. During aging, alterations in collagen homeostasis contribute to structural and functional decline of tissues [7], which in turn impair cell–matrix interactions and mechanical signaling, thereby promoting age-related phenotypes such as skin wrinkling, osteoporosis, joint degeneration, and vascular stiffness [8]. Therefore, collagen-derived products have attracted increasing attention for their potential role in health maintenance and anti-aging applications.

In this context, foods derived from collagen-rich tissues have been considered effective supplements to mitigate the loss or damage of collagen and other ECM components. Collagen-derived products are commonly consumed in the form of gelatin or collagen peptides, which are typically obtained through thermal processing or enzymatic hydrolysis. These distinct preparation methods generate products with different molecular characteristics, thereby influencing their biological activities [9,10,11]. Compared with enzymatic hydrolysis, research on the biological activity of thermally degraded collagen products remains limited, particularly in vivo. Moreover, the digestion and absorption of thermally derived products largely depend on individual gastrointestinal proteolytic capacity, resulting in considerable variability in the generation and bioavailability of functional peptides [12]. In contrast, enzymatic hydrolysis enables the controlled production of low-molecular-weight collagen peptides with defined compositions and improved bioavailability [13,14,15,16,17]. With advances in food processing technologies and increasing demand for functional foods, enzymatically derived collagen peptides have been widely developed and applied. Enzymatically derived collagen peptides have demonstrated more consistent antioxidant, anti-inflammatory, and tissue-protective effects [14,18,19], leading to their extensive use in products targeting skin health, bone metabolism, and aging-related disorders [20,21].

Besides the abundant sources of gelatin from mammalian species, the use and demand for collagen or gelatin from non-mammalian species is growing. Among various collagen sources, fish-derived collagen has attracted growing attention due to its high availability, favorable safety profile, and unique physicochemical properties [17,22,23]. Compared with mammalian collagen, fish collagen typically exhibits lower a denaturation temperature and a looser molecular structure, which facilitate processing and enzymatic hydrolysis [17,24]. Moreover, fish collagen peptides are rich in glycine, proline, and hydroxyproline residues, which have been closely associated with antioxidant and tissueprotective activities [25,26]. Importantly, fish collagen is commonly obtained from aquatic processing by-products, offering significant advantages in terms of resource sustainability and environmental impact [24,27]. Despite extensive research on the bioactivities of enzymatically hydrolyzed fish collagen peptides, far less attention has been paid to collagen-derived products from thermal processing, a matrix that more closely represents real dietary intake. Whether gelatin–peptide complexes produced by thermal degradation exhibit biological activities comparable to those generated by enzymatic hydrolysis remains largely unexplored. Addressing this question is critical for objectively evaluating the functional significance of collagen processing methods from both nutritional and industrial perspectives. To the best of our knowledge, this is the first study to systematically compare the biological and anti-aging activities of gelatin–peptide complexes derived from sturgeon skin prepared via thermal processing and enzymatic hydrolysis under a D-galactose-induced aging model.

Sturgeon (*Acipenser* spp.), an ancient lineage of fish with high economic value, provides an ideal model for addressing this issue. Global sturgeon aquaculture is driven primarily by caviar production, during which the skin and other collagen-rich tissues are generated as low-value by-products [19,28]. Previous studies have shown that collagen and collagen peptides derived from sturgeon skin exhibit notable antioxidant activities in vitro [28]. Despite these recognized biological benefits, the physiological efficacy of collagen and its peptides is strongly influenced by the form in which collagen is consumed. However, systematic comparisons between thermally processed gelatin–peptide complexes and enzymatically hydrolyzed collagen peptides derived from the same raw material are still lacking.

Therefore, in the present study, sturgeon skin obtained as a by-product of caviar pro-duction was used as the collagen source to prepare a thermally processed gelatin–peptide complex (GPC-TD) and an enzymatically hydrolyzed gelatin–peptide complex (GPC-EH). The antioxidant and anti-aging activities of these two preparations were systematically compared using cell models and a D-galactose-induced mouse aging model. This work aims to provide theoretical support for the high-value utilization of sturgeon skin resources in both food industry applications and dietary practices.

## 2. Materials and Methods

### 2.1. Materials

Frozen hybrid sturgeon skin was purchased from Zhejiang Sturgeon Dragon Aquatic Products Technology Co., Ltd. (Quzhou, China). Pepsin, trypsin, and chymotrypsin were obtained from Beijing Solabao Technology Co., Ltd. (Beijing, China). Rat adrenal chromaffin cell line (PC12) was sourced from our laboratory. Assay kits of SOD, GSH-Px, CAT, MDA, ACh, and AChE were obtained from Nanjing Jiancheng Bioengineering Institute (Nanjing, China). Fetal bovine serum was obtained from Shanghai Kanglang Biological Technology Co., Ltd. (Shanghai, China). Trypsin-EDTA solution (0.25%) and RPMI-1640 medium were purchased from Thermo Fisher Scientific (Waltham, MA, USA). Thiazolyl blue tetrazolium bromide (MTT) was purchased from Tianjin Damo Chemical Reagent Factory (Tianjin, China), and D-galactose (analytical grade) was obtained from Shanghai Macklin Biochemical Technology Co., Ltd. (Shanghai, China).

### 2.2. Preparation of Collagen Peptide Complexes (GPCs)

#### 2.2.1. Preparation of GPC-TD

Frozen hybrid sturgeon skin was washed, cut into small pieces, and pretreated with 0.25 mol/L HCl solution at a solid-to-liquid ratio of 1:10, followed by magnetic stirring for 6 h. After washing with deionized water to neutral, thermal extraction was carried out at a solid-to-liquid ratio of 1:20 at 60 °C and 121 °C for 1 h each. The resulting supernatant was collected by centrifugation, dialyzed for 24 h, and was freeze-dried. The product obtained after treatment at 121 °C was centrifuged, and the resulting supernatant was designated as GPC-TD [29].

#### 2.2.2. Preparation of GPC-EH

The freeze-dried GPC-TD powder obtained from 60 °C thermal extraction was reconstituted with deionized water at a solid-to-liquid ratio of 1:20. Enzyme hydrolysis was performed using different proteases at an enzyme dosage of 2500 U/g: neutral protease for 0.5 h, pepsin for 9 h, and a combined pepsin-trypsin system for 2.5 h. After hydrolysis, the enzymes were inactivated by boiling for 20 min, the pH was adjusted to 7.0, and the supernatant was collected by centrifugation. The solution was dialyzed for 24 h and was freeze-dried to obtain the enzymatic hydrolysis gelatin–peptide complex (GPC-EH) powder [29].

### 2.3. Cell Culture and Treatment

PC12 cells were grown in RPMI-1640 medium supplemented with 10% horse serum, 5% heat-inactivated fetal bovine serum, 100 U/mL penicillin, and 100 µg/mL streptomycin. The cultures were incubated at 37 °C in a humidified atmosphere with 5% CO_2_. Cells in the logarithmic growth phase were subsequently collected for further experiments [30].

### 2.4. Effect of D-Galactose on PC12 Cell Viability

PC12 cells at the logarithmic growth stage were seeded into 96-well plates at a density of 1.0 × 10^4^ cells per well and cultured for 24 h at 37 °C in a 5% CO_2_ atmosphere. Following attachment, different concentrations of D-galactose (200, 300, 400, 500, 600, 700, 800, and 900 mmol/L) were applied for 4 h. Each concentration was tested in quintuplicate, and the experiment was repeated at least three times. Cell viability was assessed using the MTT assay. Following treatment, 20 µL of MTT solution (5 mg/mL) was added to each well, and the cells were incubated for 4 h at 37 °C in the dark. The supernatant was then removed, and 150 µL of DMSO was added and incubated for 4 h to dissolve the formazan crystals. Absorbance was measured at 570 nm using a microplate reader(Tecan Group Ltd., Männedorf, Switzerland). Cell viability was calculated using the following formula [31]:

Viability (%) = (A_570 nm_-treatment/A_570 nm_-control) × 100%

### 2.5. Protective Effects of GPC-TD and GPC-EH Against D-Gal-Induced PC12 Cell Damage

PC12 cells in the logarithmic growth phase were seeded in 96-well plates and cultured for 24 h. Subsequently, varying concentrations of GPC-TD or GPC-EH were added, and cells were incubated for another 24 h. The tested concentrations were as follows:

GPC-TD and GPC-EH obtained by combined hydrolysis with pepsin and trypsin: 12.5, 25, 50, 100, 200, 400, 600, 800, and 1000 µg/mL

Neutral protease or pepsin hydrolyzed GPC-EH: 200, 400, 600, 800, and 1000 µg/mL

For the control and model groups, an equal volume of complete medium was added.

Except for the control group, all other groups were treated with 100 µL of 300 mmol/L D-gal for 4 h to induce oxidative damage; the control group received an equal volume of complete medium [32]. After treatment, cell viability was measured using the MTT assay as described in Section 2.4.

### 2.6. Animals and Treatment

A total of 90 healthy, specific pathogen-free female ICR mice (4 weeks old, 26 ± 2 g) were purchased from Liaoning Changsheng Biotechnology Co., Ltd. (Dalian, China). The mice were housed under controlled conditions: temperature 23 ± 2 °C, relative humidity 55–75%, and a 12 h light/dark cycle, with free access to food and water. After 14 days of adaptive feeding, the mice were randomly divided into 8 groups (*n* = 11 per group):

Blank control (saline), model (D-galactose only), and three dose levels (100, 200, and 400 mg/kg BW) of GPC-TD and GPC-EH, respectively. During the first two weeks, mice received daily gavage of the corresponding sample or saline. From weeks 3 to 10, all groups except the blank control received daily intraperitoneal injections of D-galactose (500 mg/kg) in addition to continued gavage [33]. The blank control received saline for both gavage and injection. The experiment lasted 10 weeks, with body weight measured weekly to adjust dosages. All experimental procedures were approved by the Animal Ethics Committee of Dalian University of Technology (Ethics No. DLPU2023040). Mice were observed daily for general health. At the end of the experiment, animals were anesthetized with ether in a closed chamber until loss of reflexes, followed by euthanasia via cervical dislocation. Tissues including the brain, heart, liver, spleen, stomach, kidneys, and skin were immediately excised, rinsed in cold phosphate-buffered saline (PBS) to remove blood, gently blotted, and weighed. Brain tissues were carefully dissected to avoid mechanical damage. All procedures were conducted in accordance with institutional guidelines for the care and use of laboratory animals.

### 2.7. Behavioral Assessment

#### 2.7.1. Morris Water Maze Test

The water maze tank was filled with warm water, and training trials were conducted during the first five days. During the training period, mice were guided to swim to the goal platform, and escape latency was recorded. On day 6 (memory test day), each mouse was placed at the maze exit for familiarization for approximately 1 min. Subsequently, the mouse was placed at the entrance to start the test. Each mouse was allowed a maximum of 90 s to swim. In the memory test, a computer-based analysis system(Tsinghua Tongfang, Beijing, China) recorded the time taken to reach each final platform [33].

#### 2.7.2. Nesting Behavior Test

Mice were individually housed in separate cages and allowed to acclimate to a dark environment for 24 h. After acclimation, equal amounts of bedding were added to each cage, and four sheets of unscented paper towels were laid evenly in the cage. The nesting behavior of the mice was observed using a double-blind scoring method, with a total observation time of 24 h. Nesting was scored every 8 h, based on a method adapted from the literature [34]. The specific scoring criteria are shown in Table 1.

### 2.8. Organ Index

Samples of skin, heart, liver, spleen, stomach, kidneys, and brain were excised, thoroughly rinsed with cold PBS to remove blood, and gently dried with filter paper. The organs were then weighed, and the organ index was calculated as follows [35]:

Organ index = organ weight (g)/body weight (g)

### 2.9. Biochemical Analysis

Portions of the liver and brain tissue were placed in RNase/DNase-free tubes, quickly snap-frozen in liquid nitrogen, and stored at −80 °C. Homogenates were prepared at a concentration of 10% (*w*/*v*, 100 mg tissue per 1 mL cold PBS). After homogenization on ice, the samples were centrifuged at 12,000× *g* for 10 min at 4 °C, and the supernatants were collected for analysis [36]. The activities of SOD, CAT, GSH-Px and MDA content in liver, as well as the acetylcholine (ACh) content and acetylcholinesterase (AChE) activity in brain tissue, were measured according to the manufacturer’s instructions provided in the assay kits. All enzyme activity results were standardized to total protein content (expressed as U/mg protein or nmol/mg protein, depending on the assay kit units). Protein concentration was determined using the BCA assay.

### 2.10. Masson’s Trichrome Staining

For histological analysis, tissue samples were immediately fixed in 4% paraformaldehyde solution at 4 °C for 24 h. After fixation, tissues from the skin, heart, liver, spleen, stomach, kidneys, and brain were subjected to routine procedures of dehydration, paraffin embedding, sectioning, and staining. Tissue sections were dewaxed, rehydrated and stained with Masson’s trichrome. The sections were stained for 10 min, rinsed with distilled water, dehydrated through a graded ethanol series, cleared in xylene, and mounted with coverslips for microscopic observation. Stained sections were observed under an optical microscope to evaluate collagen fiber deposition and tissue damage in each group of mice. Masson’s trichrome staining clearly differentiates tissue components: cell nuclei and collagen fibers appear blue, whereas muscle fibers, smooth muscle, red blood cells, and cytoplasm appear red. All tissue sections were photographed under the same microscope settings to ensure comparability [37,38,39].

### 2.11. Nissl Staining

Brain tissues were fixed in 4% paraformaldehyde at 4 °C for 24 h, followed by dehydration through a graded ethanol series and embedding in paraffin. Coronal sections (5 µm) were prepared using a microtome and mounted on glass slides. The sections were deparaffinized in xylene, rehydrated through a descending ethanol series, and rinsed in distilled water. Nissl staining was performed by immersing the sections in 0.5% toluidine blue solution for 10 min at room temperature. After staining, the sections were rinsed briefly in distilled water, differentiated in 95% ethanol for 30 s to remove background staining, and dehydrated through ascending ethanol concentrations. The slides were cleared in xylene and coverslipped using a mounting medium. Stained neurons were visualized under a light microscope(Leica Microsystems, Wetzlar, Germany), and representative images were captured for analysis [40].

### 2.12. Statistical Analysis

All experiments were conducted in triplicate, and results are presented as the mean of three independent experiments. Data from the Morris water maze test, which did not follow a normal distribution, are expressed as median values, whereas other results are reported as mean ± standard deviation (SD). Statistical analyses were performed using SPSS 23.0 (SPSS Inc., Chicago, IL, USA). Data normality and variance homogeneity were evaluated using the Shapiro–Wilk and Levene’s tests, respectively. For datasets meeting the assumptions of normality and homogeneity of variance, one-way analysis of variance (ANOVA) followed by Tukey’s post hoc test was employed. Otherwise, the Kruskal–Wallis test was applied. A *p* value < 0.05 was considered statistically significant. Figures were prepared using Origin (2024) software.

## 3. Results

### 3.1. Effect of GPC on PC12 Cell Viability

**Figure 1:**
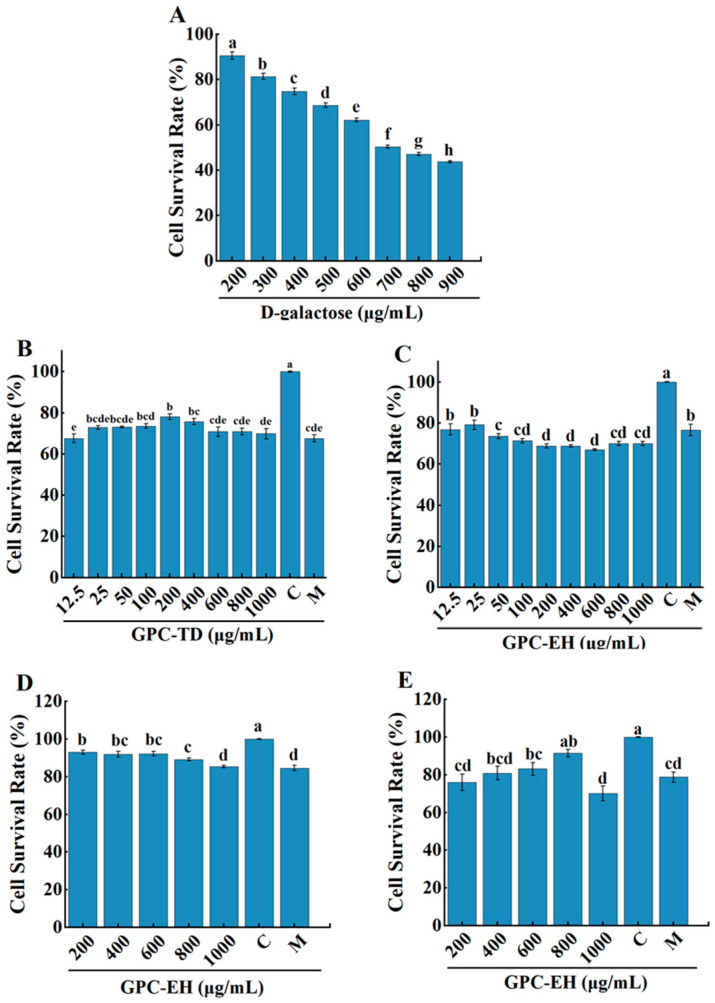
Effect of GPC on D-gal-induced injury in PC12 cells. (**A**) Effect of D-gal on viability of PC12 cells; (**B**–**E**) Effect of GPC-TD (**B**), GPC-EH derived from pepsin and trypsin (**C**), GPC-EH from neutral protease (**D**), and GPC-EH from pepsin (**E**) on D-gal-induced injure of PC12 cells. Different letters indicate significant differences within the same time period (*p* < 0.05).

Cell viability decreased in a concentration-dependent manner as D-galactose (D-gal) concentration increased from 200 to 700 mmol/L (Figure 1A). At 300 mmol/L, the level of cell damage was consistent and reproducible. Pretreatment with GPC-TD and GPC-EH alleviated D-gal-induced reduction in cell viability (Figure 1B–E). In GPC-TD and GPC-EH groups, significant protective effects were observed at relatively low doses (Figure 1D).

### 3.2. Effects of GPC on Body Weight, Organ Index, and Body Moisture

**Figure 2:**
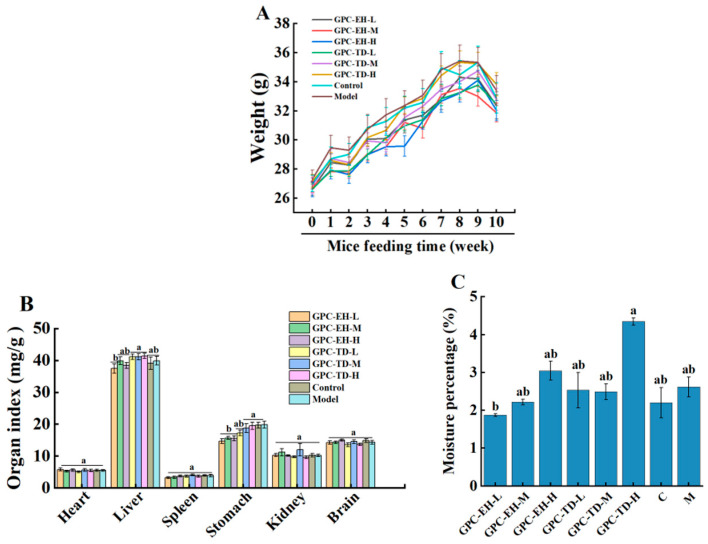
Effects of GPC on body weight (**A**), organ indices (**B**), and body moisture percentage (**C**) in mice. Different letters indicate significant differences within the same time period (*p* < 0.05).

Body weight (BW) was recorded weekly for each mouse. No significant differences were observed among groups throughout the study (Figure 2A). The blank and model groups maintained the highest BW in most weeks. GPC-TD-M and GPC-TD-H groups showed relatively faster weight gain, whereas GPC-TD-L and all three GPC-EH groups exhibited slower BW gain. Gastric indices of the latter groups were significantly lower than those of groups with higher BW (Figure 2B). No significant differences were observed among groups in heart, liver, spleen, kidney, or brain indices.

Body moisture measurements showed no significant change in the model group compared with the control group (Figure 2C). Dose-dependent increases in body moisture were observed in the GPC-EH groups, with the GPC-TD-H group exhibiting the highest moisture level.

### 3.3. Morris Water Maze Test

**Figure 3:**
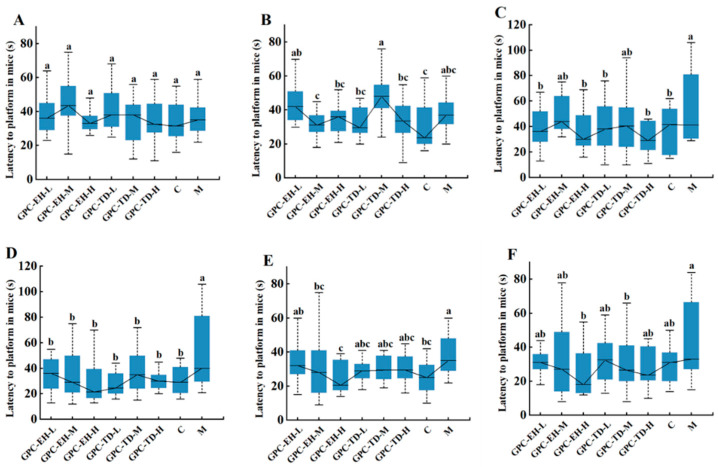
Effects of GPC on the latency for the mice to reach the platform in the Morris water maze following D-gal injection for 6 weeks. (**A**–**F**): weeks 1 through 6. Different letters indicate significant differences within the same time period (*p* < 0.05).

The latency to reach the platform was recorded over the 6-week test (Figure 3). An increase in latency was observed in the model group after two weeks of D-gal exposure (Figure 3B), and the difference reached statistical significance after three weeks (Figure 3C). At week 4, all GPC-treated groups reached the platform with latencies comparable to the control group and shorter than the model group (Figure 3D). At weeks 5 and 6, the GPC-EH-H group maintained significantly reduced latency compared with the model group, whereas no significant differences were observed for the other groups (Figure 3E,F).

### 3.4. Nesting Test

**Figure 4:**
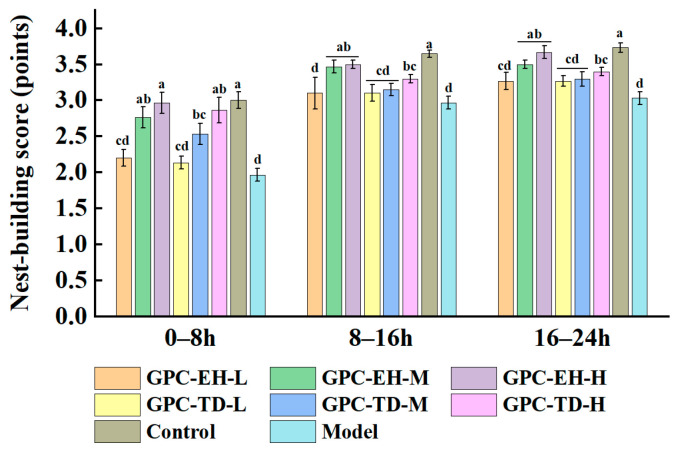
Effects of GPC on individual nest-building scores of the tested mice. Different letters indicate significant differences within the same time period (*p* < 0.05).

At the end of the study, nesting behavior was assessed over a 24 h period (Figure 4). In the early 8 h, nesting scores increased with higher doses of GPC in all treatment groups. No significant differences were observed between GPC-EH and GPC-TD groups. During the 8–16 h and 16–24 h intervals, nesting scores of the GPC-EH-M, GPC-EH-H, and GPC-TD-H groups remained significantly higher than those of the model group. Across the entire 24 h period, the GPC-EH-M and GPC-EH-H groups consistently exhibited nesting scores comparable to the control group, while only the high dose of GPC-TD group showed significantly higher scores than the model group.

### 3.5. Antioxidant Enzyme Activities and Lipid Peroxidation Levels

**Figure 5:**
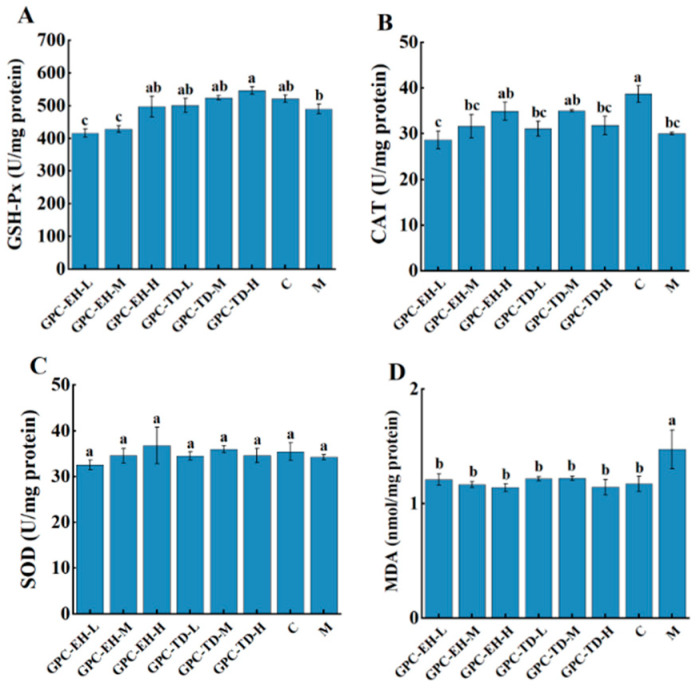
Effects of GPC on antioxidant enzyme activities and lipid peroxidation levels in liver of mice. (**A**) GSH-Px, (**B**) CAT, (**C**) SOD, (**D**) MDA. Different letters indicate significant differences (*p* < 0.05).

The activities of SOD, GSH-Px, and CAT, as well as MDA content in the liver, were measured (Figure 5). Compared with the blank group, the model group showed decreased activities of SOD and GSH-Px, while only the reduction in CAT activity reached statistical significance. In the GPC-EH groups, a dose-dependent increase in enzyme activities was observed. Only the GPC-TD-H group exhibited significantly higher GSH-Px activity than the model group (*p* < 0.05). MDA levels in all GPC-treated groups were significantly lower than those in the model group, and no significant differences were observed between these groups and the control group.

### 3.6. ACh Content and AChE Activity

**Figure 6:**
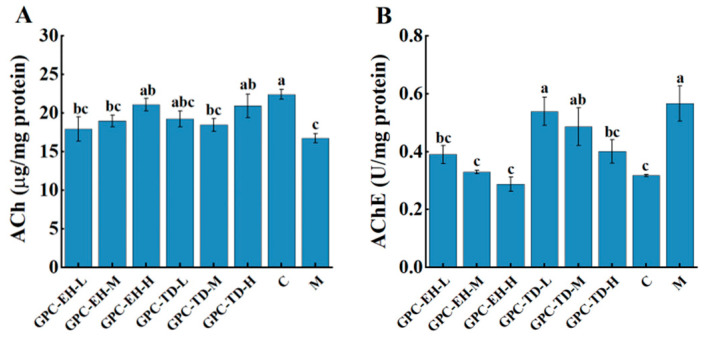
Effects of GPC-TD and GPC-EH on acetylcholine levels (**A**) and acetylcholinesterase activity (**B**) in the brains of mice. Different letters indicate significant differences (*p* < 0.05).

ACh content and AChE activity in the cerebrum were measured (Figure 6). Compared with the blank group, the model group showed a significant decrease in ACh content (Figure 6A). ACh content in the GPC-TD-H and GPC-EH-H groups was significantly higher than that in the model group. Correspondingly, AChE activity was significantly increased in the model group (Figure 6B). In the GPC-TD and GPC-EH groups, AChE activity decreased in a dose-dependent manner compared with the model group.

### 3.7. Collagen Distribution in Skin and Visceral Tissues

**Figure 7:**
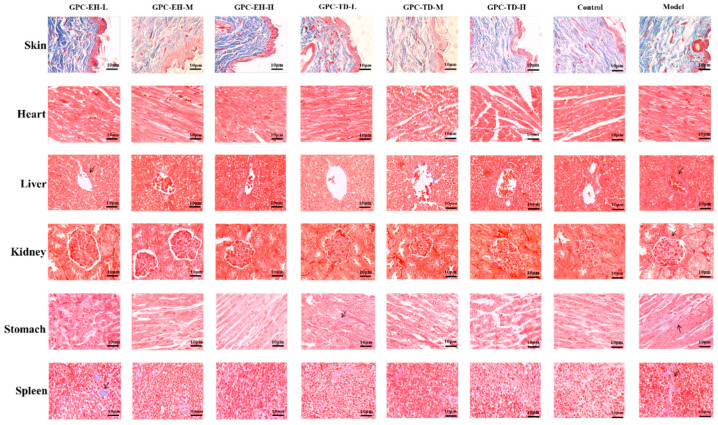
Collagen distribution and tissue morphology of mouse skin, heart, liver, kidney, stomach, and spleen by Masson’s trichrome staining. Cell nuclei and collagen fibers appear blue, whereas muscle fibers, smooth muscle, red blood cells, and cytoplasm appear red.

Masson’s staining showed collagen fibers in blue and other non-collagen components in red (Figure 7). In the model group, dermal thickness was reduced, and collagen fibers appeared irregular and unevenly distributed, whereas the blank group showed more uniform collagen arrangement. In the GPC-TD and GPC-EH groups, collagen fibers displayed increasingly organized distribution with higher doses.

In heart tissue sections, the model group exhibited collagen deposition, vacuolization in cardiomyocyte cytoplasm, and abnormal cell accumulation. Liver sections showed a disrupted radial arrangement of hepatic cords in the lobules. In the kidney, increased collagen deposition was observed in the walls of Bowman’s capsule, glomerular tufts, and arterioles. Stomach and spleen sections also exhibited collagen accumulation, with thickened muscle fibers in the stomach. These histological changes were reduced in the GPC-TD and GPC-EH groups, with dose-dependent differences observed.

### 3.8. Nissl Body Staining of Hippocampal Neurons

**Figure 8:**
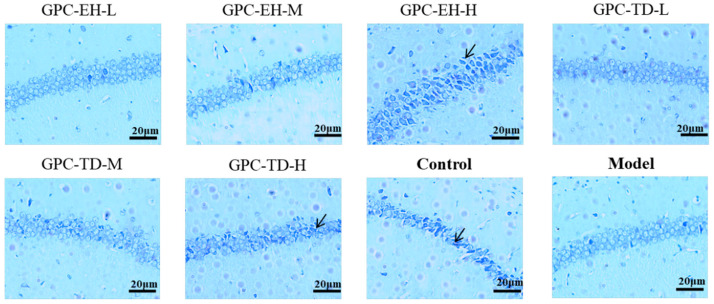
The number of Nissl bodies in the hippocampal neurons of mice by toluidine blue staining. Nissl bodies appear as blue basophilic granular or small clump-like structures in the neuronal cytoplasm.

Nissl staining of neurons was performed (Figure 8). Compared with the control group, the model group showed a marked reduction in Nissl bodies, and neuronal cytoplasm appeared pale and vacuolated. Treatment with GPC-TD and GPC-EH increased the number of Nissl bodies compared with the model group, with the GPC-EH-H group showing the highest number among the treated groups.

## 4. Discussion

Collagen peptides have been shown to scavenge free radicals, inhibit lipid peroxidation, enhance endogenous antioxidant enzyme activities (including superoxide dismutase, catalase, and glutathione peroxidase), and reduce oxidative stress markers such as malondialdehyde [19,20,28], which are considered key mechanisms mitigating oxidative damage, attenuating ECM degradation, and delaying aging-related functional decline [21].

Dietary collagen from sturgeon is obtained either through thermal processing (gelatin-based foods) or enzymatic hydrolysis (collagen peptides). While previous studies have extensively characterized the extraction processes and physicochemical properties of sturgeon gelatin [33,41,42,43] and have explored bioactivities of enzymatically hydrolyzed collagen peptides—including antioxidant [34,44,45,46,47,48,49,50], anti-inflammatory [49,51], anti-osteoarthritis [52], ACE-inhibitory [48], and blood glucose-lowering effects [53,54]—comparative investigations between thermally extracted and enzymatically hydrolyzed gelatin–peptide complexes remain lacking.

In this study, we performed the first systematic comparison of sturgeon skin gelatin–peptide complexes prepared via thermal degradation (GPC-TD) and enzymatic hydrolysis (GPC-EH) in a D-gal-induced aging model. Both GPC-TD and GPC-EH, derived from neutral protease hydrolysate, demonstrated protective effects in PC12 cells and aging mice, providing a basis for evaluating their relative biological activities in mitigating D-gal-induced cellular and organismal aging.

Under ad libitum feeding conditions, ICR mice were known to gain weight rapidly [55], and such excessive eating and rapid body weight gain may have induced gastric inflammation due to stress and minor tissue damage. Collagen peptides derived from fish have been well documented to possess significant potential in preventing and alleviating gastric mucosal edema and inflammation, attributed to their excellent antioxidant and anti-inflammatory properties [56,57]. These findings, together with the in vitro antioxidant data (Figure 1) and Masson staining results (Figure 7), may explain the ability of GPC-EH to reduce the stomach index. Furthermore, the body moisture content results (Figure 2C) also supported the notion that GPC-EH might lower the stomach index by directly eliminating inflammatory swelling and fluid accumulation. In contrast, thermal treatment may have hydrolyzed the collagen at different amino acid sites compared with enzymatic hydrolysis, accordingly, GPC-TD did not exhibit the ability to reduce the stomach index.

Aging is associated with declines in cognitive and memory functions [58,59]. The present findings supported previous reports that D-gal-induced oxidative stress and neuroinflammation impair hippocampal function and learning ability [42,46]. Our results indicated that both GPC-TD and GPC-EH mitigated these age-related cognitive declines, with GPC-EH showing slightly stronger effects. These observations are consistent with prior studies highlighting the neuroprotective and cognitive benefits of collagen peptides [43,45]. The improvement in nesting behavior further suggests that GPC administration may enhance executive function and instinctive behavioral responses, providing a complementary measure of cognitive preservation in aging models [44]. The cholinergic system, with acetylcholine (ACh) as its core neurotransmitter, is essential for cognitive function, neural signaling, and memory activities [49]. Our study suggested that GPC-TD and GPC-EH may support cholinergic function in D-gal-induced aging, potentially through antioxidant-mediated protection of neurons and/or direct modulation of AChE activity [50]. These findings provided mechanistic insight into how collagen peptides may contribute to the maintenance of cognitive performance in aging models.

Studies have shown that D-gal markedly decreases endogenous antioxidant enzyme activities (SOD, CAT, GSH-Px) [47]. In this study, however, only a significant decrease in CAT was observed in model group. Based on the data of Figure 5, antioxidant enzyme activities did not appear to be substantially affected. Nevertheless, considering the MDA results and the behavior data, there was no doubt that GPC enhanced the systemic antioxidant level in the tested mice. This suggested that GPC exerted a direct antioxidant effect rather than acting by enhancing antioxidant enzyme activity. Long-term administration of D-gal can induce structural and functional damage in multiple tissues, including ECM disorganization and fibrosis [60]. In this study, such damage was assessed using Masson’s staining. A common feature was abnormal collagen fiber deposition. Examination of tissue sections from the skin, heart, liver, kidney, stomach, and spleen revealed marked alleviation in both GPC-TD and GPC-EH groups. Both preparations reduced collagen deposition and restored tissue structure, with GPC-EH demonstrating superior efficacy. This finding was consistent with the previous report [13]. Regarding brain tissue structure, Nissl bodies are indicative of neuronal protein synthesis and metabolic activity [61]. Histological analyses revealed that neuronal integrity, as reflected by Nissl body density, was compromised in the model group. GPC-TD and GPC-EH treatment appeared to preserve neuronal morphology, with a more pronounced effect observed in the GPC-EH-H group. These neuroprotective effects were consistent with the behavioral improvements and suggested that collagen peptides may exert their cognitive benefits, at least in part, by maintaining neuronal structure and supporting cellular resilience in aging models.

Studies have shown that the mechanisms of action of D-galactose mainly include the following aspects: oxidative stress [62], mitochondrial dysfunction [63], advanced glycation end-product formation [62], neuroinflammation [64], and endoplasmic reticulum stress [65]. Collectively, these results indicated that both GPC-TD and GPC-EH derived from sturgeon skin effectively mitigated oxidative damage and tissue degeneration induced by D-gal in the aging model. These findings provided a basis for further investigations into their potential biological effects, including potential relevance to human health. As a traditional food in households and catering, gelatin jelly (containing GPC-TD) made from sturgeon skin not only offered a pleasant taste and texture, but also exhibited antioxidant even anti-aging activity. Furthermore, this study also demonstrated that GPC-EH appears slightly more effective than GPC-TD in terms of cognitive-enhancing activities. This enhanced efficacy may have been attributed to the differences in hydrolysis sites. Given that vitamin C is an essential cofactor for prolyl and lysyl hydroxylases, which are required for the post-translational modification and stable synthesis of endogenous collagen, future studies will include vitamin C in the diet to examine its impact on the differential absorption of GPC-TD and GPC-EH by the body.

## 5. Conclusions

This study systematically compared the effects of thermal degradation (GPC-TD) and enzymatic hydrolysis (GPC-EH) of sturgeon skin gelatin–peptide complexes in a D-gal-induced aging model. The results demonstrated that both GPC-TD and GPC-EH exhibited anti-aging activity, with GPC-EH showing stronger and dose-dependent effects. The key mechanisms involved enhancement of antioxidant defenses, improvement of cholinergic system function, and regulation of ECM collagen metabolism, which collectively mitigated cellular damage, cognitive decline, and tissue degeneration. Notably, this study provides novel evidence that GPC-EH can regulate the stomach index, highlighting a previously unreported aspect of its bioactivity.

Despite these significant findings, this study has limitations, including the use of a single aging model and limited investigation of long-term effects. Future research should explore the molecular mechanisms in more depth, evaluate the translational potential in human studies, and investigate the broader functional applications of GPC-EH in aging-related interventions.

Overall, these results emphasize the potent anti-aging effects of sturgeon-skin-derived GPC, underscore the novelty of GPC-EH in stomach function regulation, and provide a foundation for its development as a functional food ingredient.

## Figures and Tables

**Table 1:** Nesting experiment scoring criteria.

Scores	Nesting
1	Paper scattered, no chewing
2	Paper gathered, slight chewing
3	Paper gathered into a flat nest, noticeable chewing
4	Paper piled into a nest, small pieces chewed

## Data Availability

The original contributions presented in this study are included in the article; further inquiries can be directed to the corresponding author.
